# Earliest tea as evidence for one branch of the Silk Road across the Tibetan Plateau

**DOI:** 10.1038/srep18955

**Published:** 2016-01-07

**Authors:** Houyuan Lu, Jianping Zhang, Yimin Yang, Xiaoyan Yang, Baiqing Xu, Wuzhan Yang, Tao Tong, Shubo Jin, Caiming Shen, Huiyun Rao, Xingguo Li, Hongliang Lu, Dorian Q. Fuller, Luo Wang, Can Wang, Deke Xu, Naiqin Wu

**Affiliations:** 1 Key Laboratory of Cenozoic Geology and Environment, Institute of Geology and Geophysics, Chinese Academy of Sciences, Beijing 100029, China; 2 Center for Excellence in Tibetan Plateau Earth Science, Chinese Academy of Sciences, Beijing 100101, China; 3 University of Chinese Academy of Sciences, Beijing 100049, China; 4 Institute of Geographic Sciences and Natural Resources, Chinese Academy of Sciences, Beijing 100101, China; 5 Institute of Tibetan Plateau Research, Chinese Academy of Sciences, Beijing 100101, China; 6 Shaanxi Provincial Institute of Archaeology, Xi’an 710001, China; 7 Institute of Archaeology, Chinese Academy of Social Sciences, Beijing 100710, China; 8 Ministry of Industry and Information Technology, Beijing 100804, China; 9 Key Laboratory of Plateau Lake Ecology and Global Change, Yunnan Normal University, Kunming 650092, China; 10 Ali district culture bureau of Tibet, Ali 859000, China; 11 Center for Tibetan Studies of Sichuan University, Chengdu 610064, China; 12 Institute of Archaeology, University College London, 31-34 Gordon Square, London WC1H 0PY, U.K

## Abstract

Phytoliths and biomolecular components extracted from ancient plant remains from Chang’an (Xi’an, the city where the Silk Road begins) and Ngari (Ali) in western Tibet, China, show that the tea was grown 2100 years ago to cater for the drinking habits of the Western Han Dynasty (207BCE-9CE), and then carried toward central Asia by ca.200CE, several hundred years earlier than previously recorded. The earliest physical evidence of tea from both the Chang’an and Ngari regions suggests that a branch of the Silk Road across the Tibetan Plateau, was established by the second to third century CE.

Tea (*Camellia sinensis* L.) is one of the most popular nonalcoholic beverages, consumed by over two-thirds of the world’s population for its refreshing taste, aroma, medicinal, and mildly stimulating qualities^1^. The exact antiquity of tea is shrouded in Chinese myth^2^. The first unambiguous textual reference to the consumption of tea as a beverage can be dated to 59 BCE during the Western Han Dynasty^2^,^3^. However, its widespread popularity amongst both northern Chinese and people to the west such as Uighurs is generally attributed to the Tang Dynasty (7^th^–8^th^ century CE)^4^. Previously the oldest physical evidence of tea was from China’s Northern Song Dynasty (960–1127 CE)^5^. It has long been hypothesized that tea, silks and porcelain were key commodities exported from the ancient Chinese capital, Chang’an, to central Asia and beyond by caravans following several transport routes constituting the network commonly referred to as the Silk Road^6^,^7^,^8^,^9^,^10^, in use by the second century BCE. However, there are no records of tea having been carried along the Silk Road into Tibet, central Asia or southern Asia until the Tang Dynasty (618–907 CE)^6^,^7^. The Tibetan Plateau was then closely linked eastwards to central China through trade of tea and horses for Tibetan furs and medicinal plants^6^,^7^,^8^,^9^,^10^. Although trade of millets already connected the Tibetan Plateau to lowland China more than 4000 calibrated years before present (yr BP)^11^, and barley cultivation and pastoralism expanded after 3600 yr BP^12^, the emergence of historical patterns of commodity trade and habits of tea drinking along the Silk Road and in the Tibetan Plateau has remained poorly understood, due mainly to the poor preservation of plant leaves, and the challenge of identifying decayed tea remains in archeological samples^7^,^13^.

Here, we present evidence from calcium phytoliths (calcium oxalate plant crystals), chemical biomarkers and radiocarbon dating from dried plant bundles from two funerary sites: the Han Yangling Mausoleum^14^ in Xi’an, Sha’anxi Province; and the Gurgyam Cemetery in Ngari district, western Tibet^15^,^16^ (Fig. 1a). Large modern reference collections are used to compare and contrast microfossil morphology and biomolecular components of these ancient remains to modern standards of tea and related plant species^13^. Our study reveals that tea was drunk by Han Dynasty emperors as early as 2100 yr BP and had been introduced into the Tibetan Plateau by 1800 yr BP. This indicates that one branch of the Silk Road passed through western Tibet at that time.

## Results

### Study location

**Figure 1:**
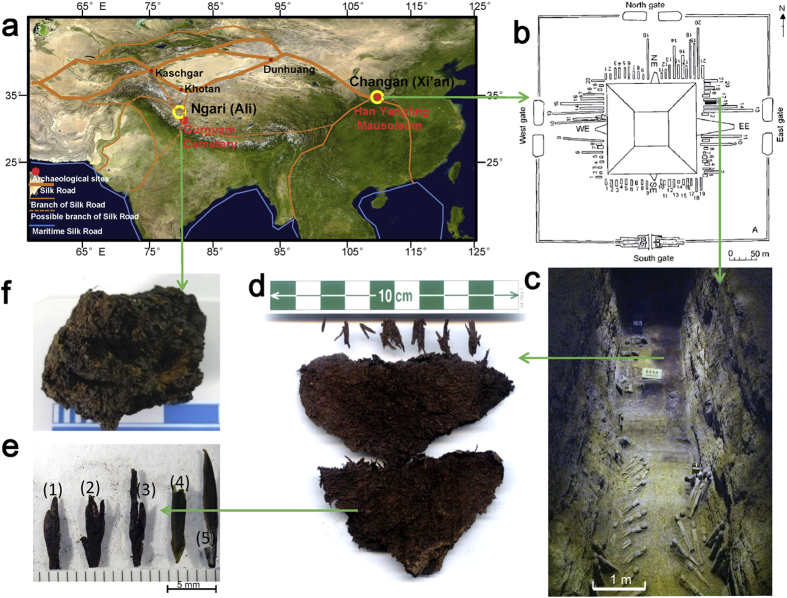
Map and photographs of early tea found in China. (**a**) Red dots show the location of the Han Yangling Mausoleum in Chang’an and Gurgyam Cemetery in Ngari, with orange lines indicating the routes of the Silk Road. (**b**) Plan of the Han Yangling Mausoleum and (**c**) the outer burial pit DK15; the brown material in pit DK15 constitutes plant remains including crops. (**d**) Sample DK15-1 taken from plant remains found in Pit DK15. (**e**) Morphological comparison between plant leaves from DK15-1 (e1, 2, 3) and modern green tea buds (e4, 5). (**f**) Sample XZ-1 taken from Gurgyam Cemetery plant remains. The firgure1a was generated using DIVA-GIS 7.5 (http://www.diva-gis.org/) and Microsoft PowerPoint 2011. The photos in Fig. 1c–f were taken by Houyuan Lu.

The Han Yangling Mausoleum (34° 26′37.99″ N, 108°56′26.84″ E, 415 m asl) is located to the north of Xi’an, on a loess platform along the north bank of the Weihe River. It was built for the Jing Emperor Liu Qi (188–141 BCE), the fourth emperor of the Western Han Dynasty, and his wife (Fig. S1a). A total of 86 outer burial pits surround the mausoleum (Fig. 1b), of which No.15 (DK15) was excavated in 1998–2005 by Shaanxi Provincial Institute of Archaeology^14^,^17^. Pit DK15 is 21 m in length and 2.6–2.7 m in width (Fig. 1c). Wooden boards divide the pit into eastern and western segments. Plant remains were noted as a large mass in the eastern part of the pit, measuring ~13 × 2 m in area and 2–8 cm in thickness and composed of various finer lamellae. These remains had partially decomposed, but includes grains and thin unconsolidated pieces colored brown to black. Some of the lamellae of crop remains have been identified by phytolith analysis and morphological features preserved macrofossils, including foxtail millet (*Setaria italica*), broomcorn millet (*Panicum miliaceum*), rice (*Oryza sativa*) and domesticated chenopod (*Chenopodium giganteum*)^14^ (Fig. S1c–e). However, one sample (DK15-1) is composed of apparent plant leaves, gathered into a dark brown brick shape (Fig. 1d, Fig. S1b). Direct AMS ^14^C dating of part of this sample has yielded ~255 ± 80 BCE (Table S1). These decomposed vegetative plant remains retained little diagnostic morphological features so taxonomic identification on this basis was not possible.

**Figure 2:**
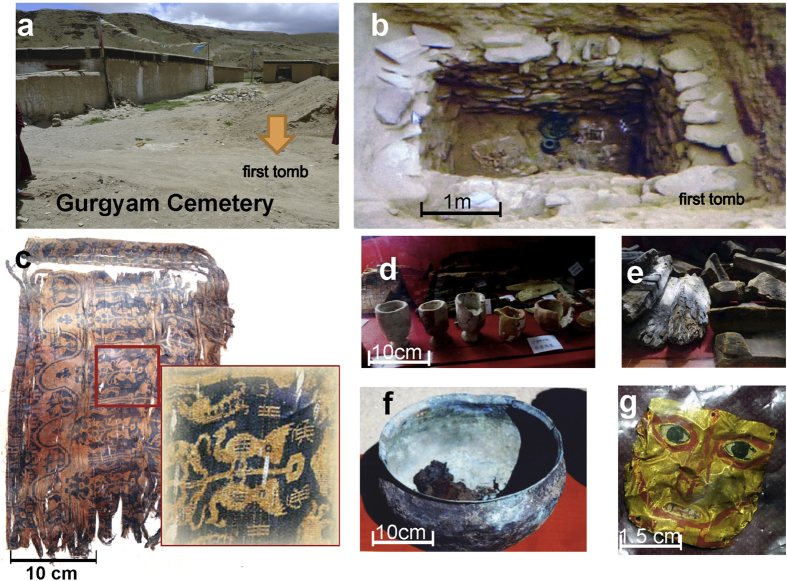
Location of the Gurgyam Cemetery and unearthed relics. (**a**) Location of the first tomb (below arrow). (**b**) The form of the first tomb. (**c**) Silk pieces with the woven Chinese characters “Wang Hou” (kings and princes). (**d**) Thick-walled, roughly-modeled ceramic beakers. (**e**) Wooden fragments. (**f**) Copper-alloy vessel. (**g**) Golden mask. The photos in Fig. 2a,d–g were taken by Houyuan Lu, and the photos in Fig. 2b,c were taken by Tao Tong.

Gurgyam Cemetery (31°7′45.08″N, 80°38′28.27″E, 4290 m asl) is located in the capital of the ancient Zhang Zhung Kingdom, on the northern upriver bank of Sutlej River (Fig. 1a) in Ngari district, Tibet. The first tomb was accidentally discovered by the monks of Gurgyam monastery in 2005 (Fig. 2a,b), and a systematic excavation was later carried out by Chinese archaeologists in 2012^15^,^16^. The tomb was buried beneath the river’s silt and gravel, with a square pit containing a square casket-shaped wooden coffin and a well-preserved skeleton. Burial artifacts include silk pieces with the woven Chinese characters “Wang Hou” (King and Princes) (Fig. 2c), various ceramic vessels, wooden tools, bronze vessels, and a golden mask (Fig. 2d–g). An unidentified object found in one ceramic vessel appears to be agglomerated plant residue. This plant residue (XZ-1) (~4×5×3 cm, Fig. 1f) and other grave goods have been dated as second to third century CE (Table S1).

### Identification of plant remains

The small plant leaves from both samples DK15-1 and XZ-1 show several morphological features that match those of tea (*e.g.* tea bud structures, Fig. 1e; Fig. S1b). However, no diagnostic morphological features survive that can be used to identify unequivocally these partially decayed leaves and buds as tea. Recently, the development of biomarker and calcium phytolith proxies has allowed the identification of components from decayed food and tea remains^2^,^13^,^18^,^19^. Further, a recent study has used both theanine and caffeine markers as a basis for tea identification^5^,^20^. Caffeine is an uncommon plant alkaloid but found across several unrelated tropical families, often prized for human consumption, including tea (Theaceae), coffee (Rubiaceae), cola and cacao (Sterculaceae), gurana (Sapindaceae), yerba mate (Aquifoliaceae), and *Citrus* flowers (Rutaceae)^21^. Theanine is an amino acid so far only reported from species in the Theaceae, especially in high levels in *Camellia sinensis*^21^,^22^. Additionally, our statistical observation of variations in calcium oxalates crystals and anatomical structures in modern tea and related Theaceae and non-Theaceae plants show that trichome bases in tea plants possess four distinctive straight and regular cracks. Indeed, *in situ* calcium phytoliths of druse form have the smallest diameter (11.65 ± 3.64 μm) in our reference collection, providing morphological criteria for distinguishing tea from other plants^13^.

In this study, we examined the biomolecular components in samples DK15-1 (Han Yangling sample) and XZ-1 (Gurgyam sample) alongside standard reference material (SRM), using ultra-performance liquid chromatography/high resolution mass spectrometry (UPLC/MS) to isolate traces of theanine (see: Methods) and gas chromatography/mass spectrometry (GC/MS) to identify traces of caffeine (see: Methods)^23^,^24^,^25^.

**Figure 3:**
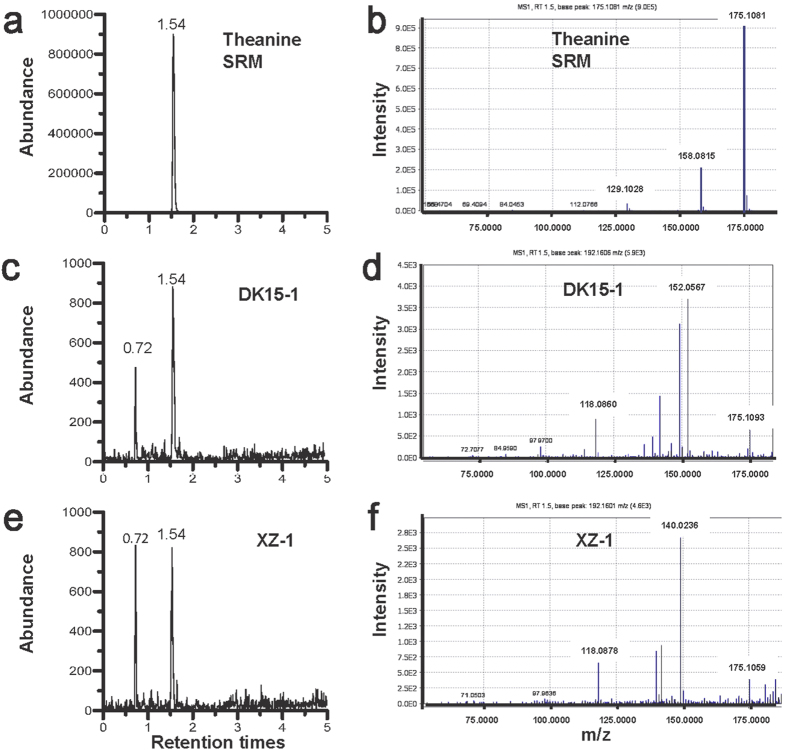
Extracted ion chromatograms and exact mass spectra for theanine from SRM theanine and archeological samples. (**a**) Ion chromatogram of SRM theanine at a 1.54 min retention time. (**b**) Exact mass spectra at m/z 175.1082 ([M + H] + ) for SRM theanine [γ-glutamylethylamide]. (**c**) Extracted ion chromatogram of Sample DK15-1, uniform with SRM theanine. (**d**) Exact mass spectra of theanine for Sample DK15-1. (**e**) Extracted ion chromatogram of Sample XZ-1, indicating theanine. (**f**) Exact mass spectrometry of theanine for Sample XZ-1.

The extracted ion chromatogram of theanine (γ-glutamylethylamide) at m/z 175.1082 ([M + H]+ , exact mass)^20^ from UPLC/MS analysis shows that the theanine peak occurs at a retention time of 1.54 min in a SRM theanine sample (Fig. 3a,b), similar to the retention times (Fig. 3c,e) of the archeological samples (DK15-1, XZ-1) (Fig. 3d,f), confirming that both archeological samples contain theanine.

**Figure 4:**
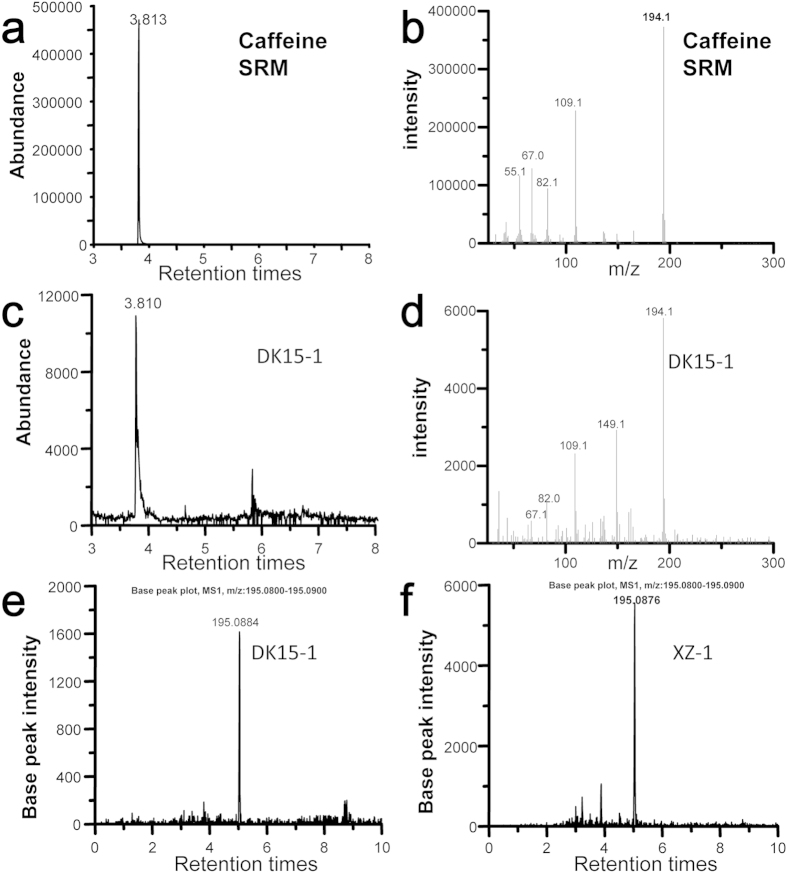
Extracted ion chromatograms and exact mass spectra for caffeine from modern tea samples and archeological samples. (**a**) Ion chromatogram for caffeine for a modern tea sample at a 3.813 min retention time, using GC/MS. (**b**) Mass spectra of caffeine [1,3,7-trimethylxanthine] for a modern tea sample, using GC/MS. (**c**) Ion chromatogram of Sample DK15-1, uniform with caffeine, and using GC/MS. (**d**) Mass spectra of caffeine for Sample DK15-1, using GC/MS. (**e**) Exact mass spectra of caffeine for Sample DK15-1, using UPLC/MS. (**f**) Exact mass spectra of caffeine for Sample XZ-1, using UPLC/MS.

By using GC/MS, caffeine (1,3,7-trimethylxanthine) exhibits a retention time of 3.813 min in both of the SRM modern tea samples (green tea) (Fig. 4a,b) and the archeological samples (DK15-1, Fig. 4c,d). Furthermore, the extracted caffeine ion chromatogram at m/z 195.0882 ([M + H] + , exact mass)^20^,^26^ using UPLC/MS analysis indicates that the caffeine peak occurs at the same retention time of 5.0 min for the archeological samples (DK15-1, XZ-1) (Fig. 4e,f), confirming that both archeological samples contain caffeine.

**Figure 5:**
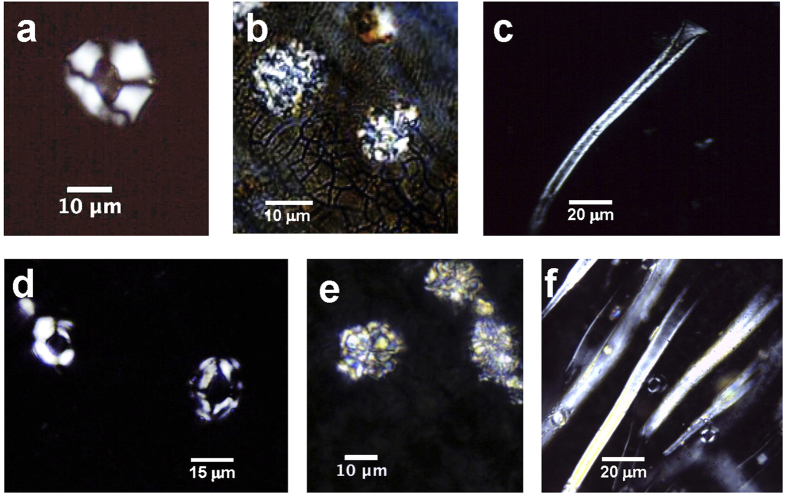
Photographs illustrating discrimination of contemporary and prehistoric tea calcium phytoliths. (**a**) Calcium phytoliths from Sample XZ-1, compared with (**d**) modern regular crack-type calcium phytoliths from trichome bases of *C. sinensis*. (**b**) Calcium phytoliths from Sample DK15-1, compared with (**e**) modern druse-type calcium phytoliths from *C. sinensis*. (**c**) Calcium phytoliths from Sample DK15-1, compared with (**f**) modern trichome type calcium phytoliths from *C. sinensis*^13^.

Therefore, the significant relative abundance of both theanine and caffeine found in the archeological samples (DK15-1, XZ-1) *vis-à-vis* similar retention times and mass fragmentation to SRM indicates that the two plant residues from the Han Yangling Mausoleum and Gurgyam Cemetery are examples of ancient tea. A further line of evidence comes from the analysis of phytoliths and calcium oxalate crystals (calcium phytoliths) of the decayed samples (DK15-1, XZ-1). Both archeological samples contain abundant calcium phytoliths, including the calcium oxalates druses and trichome base phytoliths. These calcium phytoliths also match the *genus Camellia*^13^ (Fig. 5a–f, Fig. S2a). These three diagnostic tests (for caffeine, theanine and calcium phytoliths) together confirm physical evidence for tea being imported to Xi’an in the first century BCE, and westwards into Tibet by the second century CE.

## Discussion

Traditionally, tea constitutes two or three leaves and the terminal apical buds of the tea shrub^2^,^26^. The tea buds (also known as “tips”) are the small, unopened leaves of the tea plant, and are often considered to be of better quality than the larger, older tea leaves^26^,^27^. “Imperial tea”, or that called “fine plucked”, is thus the tea bud alone and/or the two closest leaves^28^. Figures 1d,e show that the tea from Sample DK15-1 (in the mausoleum of the emperor of the Western Han Dynasty) consists almost entirely of tea buds, although the determination of tea cannot be identified simply from bud shapes (Fig. S1b). Phytolith analysis of Sample XZ-1 from Gurgyam Cemetery also reveals abundant calcium phytoliths identifiable as tea, as well as barley lemma phytoliths and unrecognizable plants (Fig. S2b–d). This indicates that the sample contains a mixture of tea, barley (*Hordeum vulgare*, Poaceae) and other plants. Therefore, it is likely that tea buds and/or leaves were consumed in a form similar to traditionally-prepared butter tea, in which tea is mixed with salt, *tsampa* (roasted barley flour) and/or ginger in the cold mountain areas of central Asia^2^,^29^. Of course, methods of brewing and consuming tea varied from culture to culture along the Silk Road^2^,^4^,^6^.

As we know, the genus *Camellia* is composed of over 110 species^30^. Among them, only one species, *C. sinensis,* is commercially used as a source of the beverage tea^31^. Exceptionally, some species of *Camellia*, e.g. *C. irrawadiensis* and *C. taliensis*, are only used for tea in specific areas in China, such as tropical South West China. *C. irrawadiensis* typically grows in upper Myanmar, and *C. taliensis* usually grows in the mountainous evergreen broad-leaved forests at altitudes from 1300 to 2700 m in southwestern Yunnan, China, and adjacent regions of northern Myanmar and Thailand^31^. It is thus highly unlikely that these species were the sources of tea in central China about 2000 years ago given their restricted geographical distribution. In addition, *C. irrawadiensis* contain very low levels of essential tea compounds, such as the caffeine content below 0.02%^31^,^32^. In our analysis, both theanine and caffeine are relatively rich, and diagnostic calcium phytoliths were found in the archeological samples, thus the species in our samples is most likely *C. sinensis* rather than another species.

Although it does not grow in Tibet, tea has traditionally played a multi-functional role in Tibetan society, as a ritual object, stimulant, and source of nutrition and medicine^29^. Up until now, the history of tea in Tibet has only been traced back to the Chinese Tang dynasty (618–907CE) and Tibetan Tubo kingdom (a kingdom located in the southeast Tibetan region that existed from the 618–842CE)^8^. It has been claimed that tea was introduced to Tibet as part of the Tang Princess Wencheng’s dowry (625–680CE) on her betrothal to the Songtsen Gambo^8^. Our findings indicate that tea, as an important component of Silk Road commerce, had been introduced to the Tibetan area by 1800 years ago, during the Zhang Zhung kingdom period^33^. This was at least four to five hundred years earlier than the the Southwest Silk Road through Yunnan which opened in the seventh century CE, and is known by historians as the “Tea Horse Road”^34^.

It is worth noting that silk materials containing the Chinese characters “Wang Hou” similar to those excavated at Gurgyam Cemetery (Fig. 2c) have also been found in Xinjiang Province. These date back to the third and fourth centuries CE^15^,^35^,^36^. Whether the silks in Gurgyam Cemetery are from eastern China or central Asia remains controversial^15^,^37^,^38^, although their presence alongside tea at Gurgyam Cemetery would suggest that they originated through trade from eastern China.

Our data indicate that the plant residues unearthed at both the Han Yangling Mausoleum and Gurgyam Cemetery are the earliest physical evidence of tea in the world. These data indicate that tea was part of trade of luxury products, alongside textiles, that moved along the Silk Road around 2000 years ago, and were traded up into Tibet. Recent archaeological work has highlighted how early intermittent exchanges between eastern China, the Tibetan Plateau and Central Asia began more than 4000 years ago, and resulted in the spread of food grains, a few fruit trees and livestock, and can be connected with the development of settled farming in the Tibetan Plateau and parts of Central Asia^11^,^12^,^39^. But later trade of the classical Silk Road was different in character, including diversified luxuries, such as silk, cotton cloth and new consumables such as tea.

## Methods

### Extraction of caffeine from modern tea

A modern tea sample (~0.2 g) was weighed, ground to powder, and transferred into a tube with 4.0 ml ethanol. The sample was then extracted under sonication for 20 min. After filtering, the extraction was evaporated and dried in a stream of N_2_. The residue was re-dissolved with 3 ml CHCl_3_/H_2_O (1:2, v/v) and subjected to further sonication for 20 min. After the sample was layered, 25 μl of the lower layer of fluid was transferred into an autosampling vial with 1 ml CHCl_3_ for GC/MS analysis.

### Extraction of caffeine from ancient samples

An ancient sample (~1 mg) was weighed, ground to powder, and transferred into a tube with 1.0 ml ethanol. The sample was then extracted under sonication for 20 min. After filtering, the extraction was evaporated and dried in a stream of N_2_. The residue was re-dissolved with 3 ml CHCl_3_/H_2_O (1:2, v/v) and subjected to further sonication for 20 min. After the sample was layered, the lower layer of fluid was transferred into an autosampling vial for GC/MS analysis.

### GC/MS analysis

GC/MS analysis was performed using a 7890A gas chromatograph and 5975C mass detector (Agilent Technologies, CA) in 70 eV electron impact (EI) mode. Analytes were separated using an Agilent HP-5MS capillary column of 30 m × 0.25 mm with a phase thickness of 0.25 μm. A 2 μl volume of the sample was injected in the splitless mode. The oven temperature program was as follows: an initial temperature of 160 °C for 0.5 min; an increase of temperature to 290 °C at a rate of 30 °C/min; and, finally, maintaining the temperature at 290 °C for 8 min. Helium was used as the carrier gas. The injector and aux-heater temperatures were set at 250 °C and 280 °C, respectively. Qualitative analysis was carried out under full-scan acquisition mode within the 30~300 u range. Compounds were identified based on the MS spectrum using NIST MS search software (version 2.0 f) and AMDIS software from the NIST 08 Mass Spectral Library database. Blank extraction was simultaneously carried out and tested using the same experimental procedure, for contamination control.

### Extraction of theanine from ancient samples

An ancient sample (10–30 mg) was weighed, ground to powder, and transferred into a tube with 5–15 ml ultrapure water. The sample was boiled for 5–10 min, subjected to sonication for 30 min at 60 °C and then centrifuged at 3,000 g for 5 min. After filtering, the extraction was evaporated to 0.5 ml under a stream of N_2_ at 80 °C. The concentrated extraction was stored at 4 °C for UPLC/MS analysis.

### Preparation of theanine standard

A standard aqueous solution of theanine (Dalian Meilun Biotech Co., Ltd, Batch No. J0820AS) was prepared with a concentration of 1 mM and stored at 4 °C for UP LC/MS analysis.

### UPLC/MS analysis

UPLC/MS was performed using a Waters ACQUITY UPLC-Xevo G2 Q-TOF mass spectrometer (Waters, USA). The chromatography was performed on a waters UPLC BEH C18 column (100 mm × 2.1 mm, 1.7 μm). The mobile phases consisted of (A) 0.1% formic acid in water and (B) 0.1% formic acid in ACN. The UPLC elution conditions were as follows: 0 min, 100% A; 1 min, 100% A; 9 min, 100% B; 11 min, 100% B; 11.1 min, 100% A; and 13 min, 100% A. The flow rate was set at 0.3 ml/min. The column was maintained at 55 °C. An injection volume of 10 μl was used for the reference standard and samples. MS analysis was performed using an electrospray ionization (ESI) source in positive mode. The desolvation gas flow rate was set to 750 l/h at a temperature of 500 °C. The cone gas was set to 25 l/h. The source temperature was 100 °C. The capillary and cone voltages were set at 3000 V and 25 V, respectively. MS spectra were acquired from m/z 50 to 1,200. An internal lock mass calibration at m/z 556.2771 with mass resolution >22,000 was used during analysis. The instrument was controlled and data were processed using MassLynx 4.1 software (Waters, USA).

### Analysis of calcium phytoliths

(1) Rehydration in 50% ethanol (30 min or longer), distilled water rinse (10–20 min), full-strength household bleach (5% sodium hypochlorite), 20 min to 2 h or longer, dependent on specimen characteristics until cleared (reasonably transparent); (2) Deionized water rinse (2 × 15 min each), thoroughly washed in deionized water and treated with 5% acetic acid to remove any calcium carbonates and phosphates, dehydration in a 50%, 70%, 95%, 100% ethanol series (10–15 min each); (3) Transitional solution of 1:1 100% ethanol : xylenes (10 min), two changes of pure xylene (10 min to indefinite storage period)^13^.

### Analysis of phytoliths

(1) Plant residue samples were placed in 20 ml of saturated nitric acid for over 12 h to oxidize organic materials completely. (2) Solutions were centrifuged at 2000 rpm for 10 min, decanted and rinsed twice with distilled water, and then rinsed with 95% ethanol until the supernatants were clear. (3) Phytolith sediments were transferred to storage vials. The residual subsamples were mounted onto microscope slides in Canada Balsam medium for photomicrography and in liquid medium for counting, measuring and line drawing. (4) Light photomicrography (phase-contrast, and microscopic interferometer) at 400× magnification was used to determine their anatomy and silicon structure patterns. (5) Phytolith parameters were measured using computer-assisted image analysis^18^,^19^.

## Additional Information

**How to cite this article**: Lu, H. *et al.* Earliest tea as evidence for one branch of the Silk Road across the Tibetan Plateau. *Sci. Rep.* **6**, 18955; doi: 10.1038/srep18955 (2016).

## Supplementary Material

Supplementary Information

## References

[b1] Karak T. & Bhagat R. M. Trace elements in tea leaves, made tea and tea infusion: A review. Food Research International 43, 2234–2252, http://dx.doi.org/10.1016/j.foodres.2010.08.010 (2010).

[b2] Harbowy M. E., Balentine D. A., Davies A. P. & Cai Y. Tea chemistry. Critical reviews in plant sciences 16, 415–480 (1997).

[b3] Guang D. X. Research on the Sichuan Manor’s Commodity Trade Economy of the Mid- Former Han Dynasty, based on Wang Pao’s “Tong Yue”. Agricultural History of China 4, 35 (2010).

[b4] Schafer E. Food In Chinese Culture: Anthropological And Historical Perspectives. 85–140 (Yale University Pres, 1977).

[b5] Fan W., Gong D., Yao Z. & Li D. Identification analysis of carbonized suspected tea from Luan tomb in the northern song dynasty Agricultural archaeology 2, 212–217 (2012).

[b6] Wang Y. F. The silk road and early foreign trade of tea in China. The tea 3, 1–3 (1988).

[b7] Wang H. R. Jingyang Fu brick tea on the Silk Road. The Silk Road 2, 31–32 (2013).

[b8] Yang F. Q. The “Ancient Tea and Horse Caravan Road,” the “Silk Road”of Southwest China. the Silk Road 2, 29–32 (2004).

[b9] Yang B. Between Winds and Clouds: The Southwest Silk Road: Yunnan in a Global Context. 1–44 (Columbia University Press, 2008).

[b10] Parzinger H. The ‘Silk Roads’ Concept Reconsidered: About Transfers, Transportation and Transcontinental Interactions in Prehistory. The Silk Road 5, 7–15 (2008).

[b11] d’Alpoim Guedes J. *et al.* Moving agriculture onto the Tibetan plateau: the archaeobotanical evidence. Archaeol Anthropol Sci 6, 255–269, 10.1007/s12520-013-0153-4 (2014).

[b12] Chen F. H. *et al.* Agriculture facilitated permanent human occupation of the Tibetan Plateau after 3600 B.P. Science 347, 248–250, 10.1126/science.1259172 (2015). 25593179

[b13] Zhang J., Lu H. & Huang L. Calciphytoliths (calcium oxalate crystals) analysis for the identification of decayed tea plants (*Camellia sinensis* L.). Sci. Rep. 4, 1–9, 10.1038/srep06703 (2014). PMC4208061 25342006

[b14] Yang X. *et al.* Plant crop remains from the outer burial pit of the Han Yangling Mausoleum and their significance to Early Western Han agriculture. Chin. Sci. Bull. 54, 1738–1743, 10.1007/s11434-009-0048-z (2009).

[b15] Jin S. B. Coming from zhang zhung 1–215 (The Tibet people’s publishing house 2012).

[b16] Tong T. The excavtion of the Gurugyam cemetery in Gar county, Ngari prefecture, Tibet Autonomous region in 2012. Acta Archaeologica Sinica 4, 564–587 (2014).

[b17] Jiao N. F. Preliminary study on outer burial pits of Han Yangling Mausoleum. Cult Relics 7, 51–57 (2006).

[b18] Piperno D. R. Phytoliths: a comprehensive guide for archaeologists and paleoecologists. 1–239 (Rowman Altamira, 2006).

[b19] Lü H. *et al.* Component and simulation of the 4,000-year-old noodles excavated from the archaeological site of Lajia in Qinghai, China. Chin. Sci. Bull. 59, 5136–5152, 10.1007/s11434-014-0663-1 (2014).

[b20] Ho C. T., Lin J. K. & Shahidi F. Tea and tea products: chemistry and health-promoting properties. 1–283 (CRC Press, 2008).

[b21] Deng W. W., Ogita S. & Ashihara H. Distribution and biosynthesis of theanine in Theaceae plants. Plant Physiology and Biochemistry 48, 70–72, http://dx.doi.org/10.1016/j.plaphy.2009.09.009 (2010). 19828327 10.1016/j.plaphy.2009.09.009

[b22] Nagata T. & Sakai S. Caffeine, flavanol and amino acid contents in leaves of hybrids and species of the section Dubiae in the genus Camellia. Japanese Journal of Breeding (Japan) 35, 1–8 (1985).

[b23] Kiehne A. & Engelhardt U. H. Thermospray-LC-MS analysis of various groups of polyphenols in tea. Z Lebensm Unters Forch 202, 48–54, 10.1007/BF01229684 (1996). 8717094

[b24] Zhao Y. *et al.* Tentative identification, quantitation, and principal component analysis of green pu-erh, green, and white teas using UPLC/DAD/MS. Food Chemistry 126, 1269–1277, http://dx.doi.org/10.1016/j.foodchem.2010.11.055 (2011). 25544798 10.1016/j.foodchem.2010.11.055 PMC4276396

[b25] Wang L., Zhao P., Zhang F., Bai A. & Pan C. Detection of caffeine in tea, instant coffee, green tea beverage, and soft drink by direct analysis in real time (DART) source coupled to single-quadrupole mass spectrometry. Journal of AOAC International 96, 353–356 (2013). 23767361 10.5740/jaoacint.12-160

[b26] Balentine D. A., Wiseman S. A. & Bouwens L. C. The chemistry of tea flavonoids. Critical Reviews in Food Science & Nutrition 37, 693–704 (1997). 9447270 10.1080/10408399709527797

[b27] Sharangi A. B. Medicinal and therapeutic potentialities of tea (Camellia sinensis L.) – A review. Food Research International 42, 529–535, http://dx.doi.org/10.1016/j.foodres.2009.01.007 (2009).

[b28] Lu W. J. Beyond the paradigm: tea-picking women in imperial China. Journal of Women’s History 15, 19–46 (2004).

[b29] Tashi N., Tang Y. W. & Zeng X. Q. Food Preparation from hulless barley in tibet. 151–158 (Springer, 2013).

[b30] Ming T. Monograph of the genus Camellia. (Yunnan Science and Technology Press, 2000).

[b31] Liu Y., Yang S. X., Ji P. Z. & Gao L. Z. Phylogeography of *Camellia taliensis* (Theaceae) inferred from chloroplast and nuclear DNA: insights into evolutionary history and conservation. BMC evolutionary biology 12, 92 (2012). 22716114 10.1186/1471-2148-12-92 PMC3495649

[b32] Nagata T. & Sakai S. Purine base pattern of *Camellia irrawadiensis*. Phytochemistry 24, 2271–2272 (1985).

[b33] Bellezza J. V. Zhang Zhung: foundations of civilization in Tibet: a historical and ethnoarchaeological study of the monuments, rock art, texts, and oral tradition of the ancient Tibetan upland. Vol. 368 1–842 (Austrian Academy of Sciences Press, 2008).

[b34] Ahmed S. & Freeman M. Pu-erh Tea and the Southwest Silk Road: An Ancient Quest for Well-Being. Herbal Gram 90, 32–43 (2011).

[b35] Kuang Y. Archeological Evidences: Embroidered Textiles of the Han and Tang Dynasties (206BC-907AD) Unearthed along the Silk Road. Asian Social Science 8, 50–54 (2012).

[b36] Zhao F. Weaving Technology. 379–493 (Springer, 2015).

[b37] Germano D. *Flight of the Khyung*. (2012). Available at: http://www.tibetarchaeology.com/april-2012. (Accessed: 27th January 2015).

[b38] Tong T. *Silks from Han to Jin Period Found near Kyung-lung dngul-mkhar, the Capital of Ancient Xiang Xiong Kingdom in Ngari, Tibet*. (2013). Available at: http://www.kaogu.net.cn/en/backup_new/new/2013/1026/42894.html. (Accessed: 1th January 2015).

[b39] Boivin N., Fuller D. Q. & Crowther A. Old World globalization and the Columbian exchange: comparison and contrast. World Archaeology 44, 452–469 (2012).

