# Supplementary material for: Earliest tea as evidence for one branch of the Silk Road across the Tibetan Plateau
Source: Sci Rep. 2016 Jan 7;6:18955. doi: 10.1038/srep18955 (PMC4704058; doi:10.1038/srep18955)
Supplement: Supplementary Information [file srep18955-s1.doc]

Supplementary Information for

**Earliest tea as evidence for one branch of the Silk Road across the Tibetan Plateau**

Houyuan Lu*, Jianping Zhang, Yimin Yang, Xiaoyan Yang, Baiqing Xu, Wuzhan Yang , Tao Tong，Shubo Jin, Caiming Shen, Huiyun Rao, Xingguo Li, Hongliang Lu, Dorian Q Fuller, Luo Wang, Can Wang, Deke Xu, Naiqin Wu

*Corresponding author. E-mail: [houyuanlu@mail.iggcas.ac.cn](mailto:houyuanlu@mail.iggcas.ac.cn) (H.Y.Lu)

This Supplementary Text file includes:

Figs. S1 to S2

Table S1


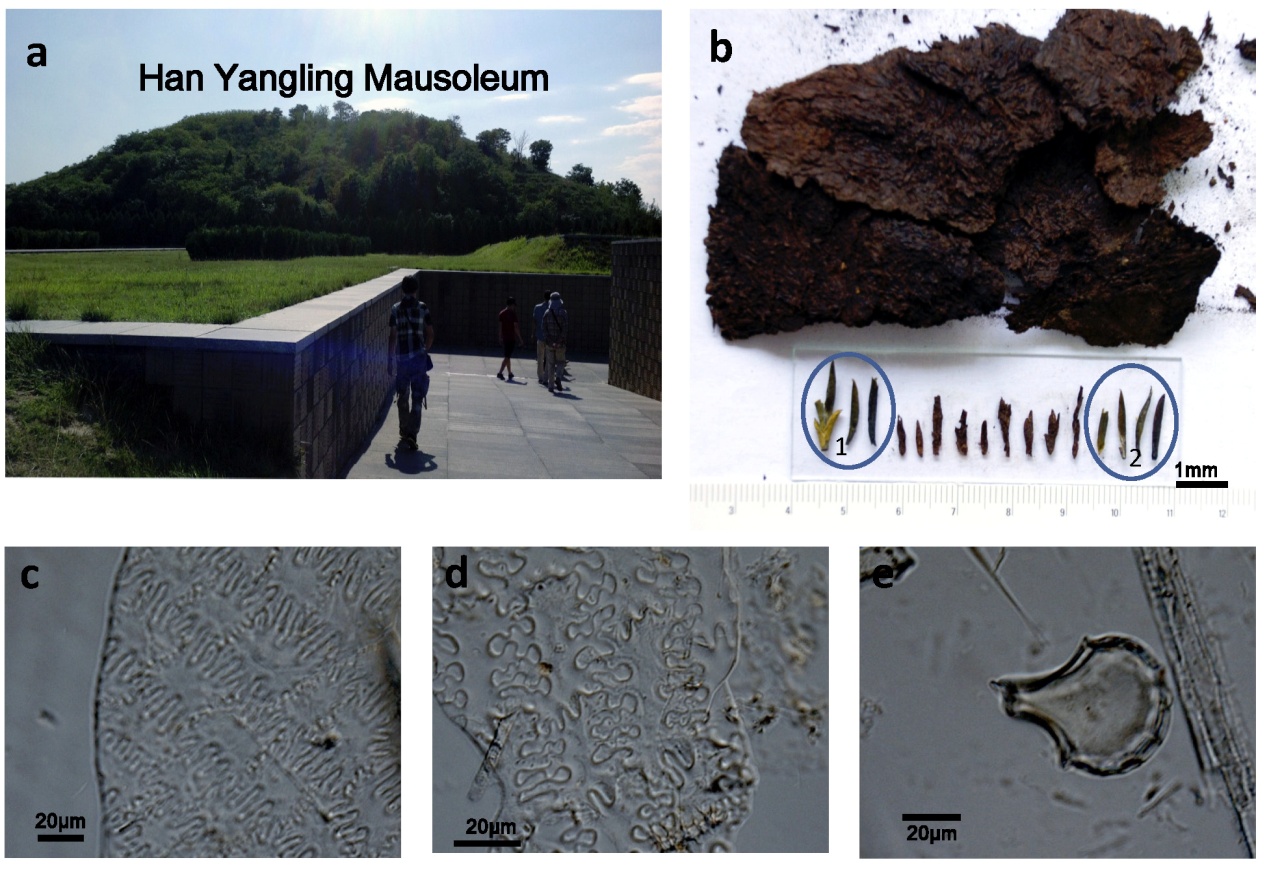


**Figure S1 Han Yangling Mausoleum and unearthed crops.**

(**a**) Perspective of the Han Yangling Mausoleum. (**b**) The DK15-1 sample is composed of decayed plant leaves (top half); morphological comparison between plant leaves from Sample DK15-1 (lower middle part) and modern green tea buds (blue circle 1, blue circle 2, respectively). (**c**) Phytoliths from broomcorn husks found in plant remains at Pit DK15. (**d**) Phytoliths from foxtail millet husks found in plant remains at Pit DK15. (**e**) Phytoliths from rice leaves and stems found in plant remains at Pit DK15. The photo in figure S1a were taken by Houyuan Lu


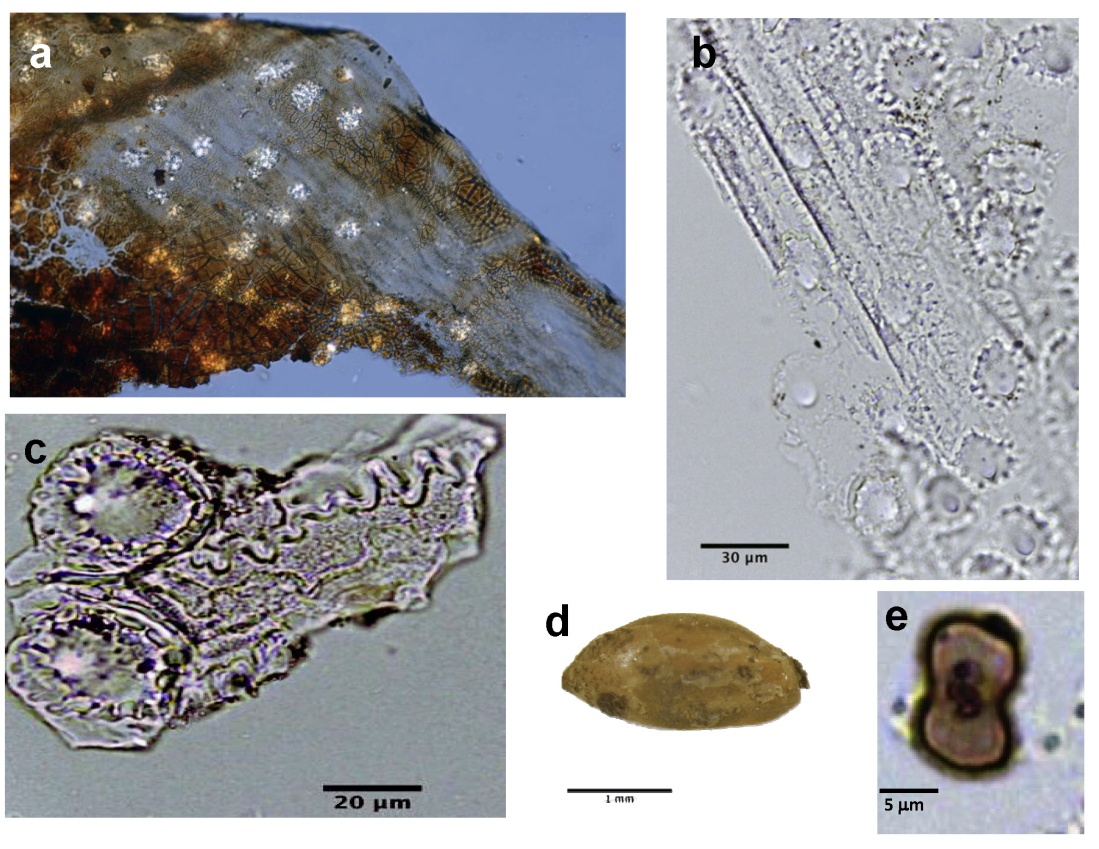


**Figure S2 Phytoliths and macrofossils found in the plant remains.**

(**a**) Calci-phytoliths from the buds of plant remains found in Sample DK15-1 at the Han Yangling Mausoleum. (**b**) and (**c**) Phytoliths from highland barley husks found in sample ZX-1. (**d**) Unidentified plant seeds from Sample ZX-1. (**e**) Phytoliths from gramineous leaves of found in Sample ZX-1.

**Table S1 ^14^C dating and calendar calibration of plant samples excavated from the Han Yangling Mausoleum and Gurgyam Cemetery sites**

| Lab. No. | Site | No. | Material age | Radiocarbon  age (yr B.P.) | Calibrated age (cal yr B.P.) | | Calendric Age cal AD/BC |
| --- | --- | --- | --- | --- | --- | --- | --- |
|  |  |  |  |  | Calendric Age calBP | 68% range calBP |  |
| **BA-121472** | GC | 12GGM1 | wood | 1855±25 | 1791 ± 41 | 1749 - 1832 | 159 ± 41 calAD |
| **BA-121473** | GC | M2-charcoal-1 | charcoal | 1830±30 | 1772 ± 39 | 1733 - 1811 | 178 ± 39 calAD |
| **BA-121474** | GC | M2-charcoal-2 | charcoal | 1795±30 | 1731 ± 58 | 1673 - 1789 | 219 ± 58 calAD |
| **BA-121477** | GC | M4-2 | Wood- bark | 1855±25 | 1791 ± 41 | 1749 - 1832 | 159 ± 41 calAD |
| **BETA-354599** | GC | XZ-1 | plant materia | 1700 ± 30 | 1624 ± 53 | 1571 - 1677 | 326 ± 53 calAD |
| **XA-5475** | GC | S | Silk | 1747 ± 23 | 1662 ± 35 | 1627 - 1697 | 288 ± 35 calAD |
| **BETA-350380** | HY | DK15-1 | plant materia | 2160 ± 30 | 2205 ± 80 | 2125 - 2285 | 255 ± 80 calBC |

**Notes:**

GC= Gurgyam Cemetery; HY= Han Yangling Mausoleum

BA=Laboratory of Peking University Accelerator Mass Spectrometry

BETA = Beta Analytic Radiocarbon Dating

XA= Xi 'an Accelerator Mass Spectrometry Center

Calendar age calibration is performed using the IntCal13 calibration curve^1^.

References

1. Reimer *PJ, et a*l. IntCal13 and Marine13 Radiocarbon Age Calibration Curves 0–50,000 Years cal BP*. Radiocarb*o**n** 55, 1869-1887 (2013).
